# On Hydrocephalus

**Published:** 1807-09

**Authors:** 


### *To the Editors of the Medical and Physical Journal.*

GENTLEMEN,

TWO cases have lately occurred within the circle of my own observation, attended with the usual symptoms of "Hydrocephalus Internus." The subjects were both under two years of age, and as the disease assumed the same appearance in both, I shall not encroach on your valuable pages by a tedious description of each individually, but lay before you the treatment adopted in both instances.

They felt an acute pain in the head, nape of the neck, and across the forehead; respiration became difficult and irregular; pulse slow, and fluttering; the optic nerve paralytic, and the iris immovable. As the disease originates in a deficiency of the absorption of the fluid in the ventricles of the brain, whatever tends to restore the necessary absorption must of course undermine the disease, and be conducive to the relief of the patient. With this intention, powders composed of calom. ppt. and pulv. antimon. were given every four or five hours; and ℈j of ungt. hydrarg. fort. directed to be rubbed into each arm or thigh every night. A blister was applied to the nape of the neck, and mercurial purges given every second or third morning. By strictly adhering to this plan they gradually began to amend; the pulse and respiration became more natural, the pains by degrees subsided, the optic nerve performed its usual functions, and the iris regained its former motion.

Dr. Cullen, to whose indefatigable researches the medical world are particularly indebted, places the internal "hydrocephalus" as a species of apoplexy — "Apoplexia Hydrocephalica;" and the late Dr. Whytt strenuously insisted, that in cases of hydrocephalus the fluid was contained on the outside of the brain; and once went so far (to satisfy his mind with respect to the propriety or fallacy of his theory) as to have a puncture made; when having cut through the dura mater, a probe was introduced without any discharge of water; which is, in my opinion, a sufficient proof of the fluid having been contained in the ventricles of the brain.

